# AI-assisted literature exploration of innovative Chinese medicine formulas

**DOI:** 10.3389/fphar.2024.1347882

**Published:** 2024-03-22

**Authors:** Meng-Chi Chung, Li-Jen Su, Chien-Lin Chen, Li-Ching Wu

**Affiliations:** ^1^ Department of Biomedical Science and Engineering, National Central University (NCU), Jhong-Li City, Taiwan; ^2^ Education and Research Center for Technology Assisted Substance Abuse Prevention and Management, National Central University (NCU), Taoyuan, Taiwan; ^3^ Core Facilities for High Throughput Experimental Analysis, Department of Biomedical Sciences and Engineering, National Central University (NCU), Taoyuan, Taiwan; ^4^ IIHMED Reproductive Center, Taipei, Taiwan; ^5^ Tian Medicine Phamaceutical Company Ltd., Taipei, Taiwan; ^6^ School of Post-Baccalaureate Chinese Medicine, Tzu Chi University, Hualien, Taiwan; ^7^ Department of Health Promotion and Health Education, National Taiwan Normal University, Taipei, Taiwan

**Keywords:** text annotation tool, TCM, text mining, extraction, TCM LSTM generative model

## Abstract

**Objective:** Our study provides an innovative approach to exploring herbal formulas that contribute to the promotion of sustainability and biodiversity conservation. We employ data mining, integrating keyword extraction, association rules, and LSTM-based generative models to analyze classical Traditional Chinese Medicine (TCM) texts. We systematically decode classical Chinese medical literature, conduct statistical analyses, and link these historical texts with modern pharmacogenomic references to explore potential alternatives.

**Methods:** We present a novel iterative keyword extraction approach for discerning diverse herbs in historical TCM texts from the Pu-Ji Fang copies. Utilizing association rules, we uncover previously unexplored herb pairs. To bridge classical TCM herbal pairs with modern genetic relationships, we conduct gene-herb searches in PubMed and statistically validate this genetic literature as supporting evidence. We have expanded on the present work by developing a generative language model for suggesting innovative TCM formulations based on textual herb combinations.

**Results:** We collected associations with 7,664 PubMed cross-search entries for gene-herb and 934 for Shenqifuzheng Injection as a positive control. We analyzed 16,384 keyword combinations from Pu-Ji Fang’s 426 volumes, employing statistical methods to probe gene-herb associations, focusing on examining differences among the target genes and Pu-Ji Fang herbs.

**Conclusion:** Analyzing Pu-Ji Fang reveals a historical focus on flavor over medicinal aspects in TCM. We extend our work on developing a generative model from classical textual keywords to rapidly produces novel herbal compositions or TCM formulations. This integrated approach enhances our comprehension of TCM by merging ancient text analysis, modern genetic research, and generative modeling.

## Graphical Abstract

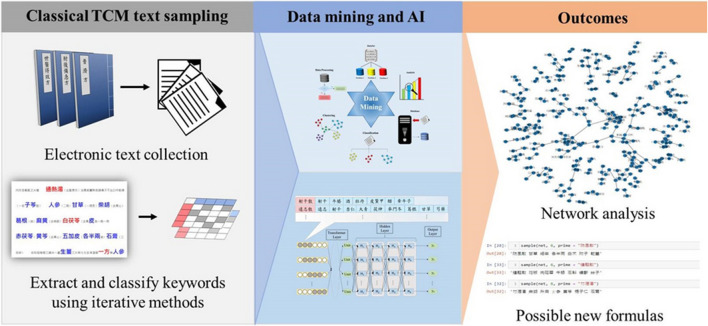

## 1 Introduction

With over 2,500 years of history, Traditional Chinese Medicine (TCM) is a renowned ancient medical system (Cheung, 2011); historical medical reports detailing herbal and animal treatments in use enrich its wisdom (Li et al., 2008). These texts have modern medical significance (Feng et al., 2006), as seen in the case of artemisinin, a life-saving anti-malarial derived from TCM (Tu, 2016). TCM remedies often blend multiple herbs or animals into formulas (Sucher, 2013), a central therapeutic approach (Chen et al., 2019) practiced for millennia. The burgeoning demand for specific flora and fauna, fueled by the utilization of widely practiced Chinese medical formulas, constitutes a compelling ecological and conservation issue in scholarly conversations (Wang et al., 2022). This trend reflects a growing reliance on traditional remedies and underscores the urgency of addressing the resultant threats to biodiversity and ecosystem stability, thereby underscoring the pressing need for comprehensive research and conservation strategies in this area (Cheung et al., 2021; Moorhouse et al., 2021; Wang et al., 2022). However, TCM popularity threatens biodiversity (Byard, 2016), urging the identification of substitute herbs with equivalent effects. Global wildlife trade affects approximately 24% of the world’s diverse vertebrate species, numbering in the tens of thousands (Scheffers et al., 2019). Reports on the wildlife trade reveal that the documented international trade rate, comprising 59%, exceeds the corresponding domestic trade rate of 41%. Within this, around 41% is for high-value commodities and food. Furthermore, traditional medicine typically utilizes up to 25% of herbal medicines (Rough Trade, 2013).

Additionally, a May 2023 report highlights a changing perception of Chinese medicine, suggesting that certain foods benefit individual health and wellbeing due to their intrinsic properties that either support or counterbalance the body’s internal equilibrium (Chen, 2023). With the above two pieces of information, the nuanced interplay between traditional medicinal practices and dietary components could surpass the reported ratio of 25% for Chinese medicine usage.

Following the above evidence, the ecological balance confronting wild animals demands a broader consideration. Specific species, such as rhinoceros horn (Still, 2003), deer musk (Liu K. et al., 2021), and donkey skin (Kubo and Zhao, 2022), benefit from protective measures within their native countries, strategically implemented to mitigate exploitation. However, using refined, mainly animal-derived derivatives to manufacture pharmaceutical products still raises humane concerns. Exemplifying this complex interplay is the extraction of gelatin, known as ejiao (阿膠) in traditional Chinese medicine, from donkey skin, a practice deeply rooted in Chinese herbal traditions (Kubo and Zhao, 2022). Therefore, we aim to reduce the impact of human civilization on the ecosystem by mitigating the portion related to traditional Chinese medicine.

Moreover, marking a noteworthy development, the ICD-11 incorporates, for the first time, a dedicated section on Chinese medicine, a move widely interpreted as indicative of a growing acceptance and recognition of the traditional practice within the international healthcare framework (Lam et al., 2019). Such instances underscore the intricate relationship between the burgeoning global demand for medicinal resources and the pressing imperative for comprehensive conservation efforts. Achieving a balance between global demand and conservation efforts is crucial.

Identifying substitute herbs with equivalent pharmacological effects has been an challenge task for researchers (Byard, 2016). Researchers have investigated existing formulations (Chen et al., 2019), considering multi-omics networks and computational models (Wang et al., 2021). Recent work by Xia et al. used association rules to explore potential COVID-19 therapies (Xia et al., 2021), revealing the need for a more comprehensive survey of ancient herbal pairs. Yet, few studies employ association rules from classical TCM texts. We propose an automated keyword extraction approach to discover herbal pairs in classical Chinese medical literature.

Analyzing ancient TCM texts presents challenges in technological skills and labor intensity (Zhang et al., 2022). Automated keyword extraction simplifies text comprehension by identifying key terms and classifying main concepts (Ercan and Cicekli, 2007). Machine learning and NLP aid in deciphering human language in keyword extraction (Turney, 2002), as exemplified by Jos A. Reyes-Ortiz et al. work on clinical decisions (Reyes-Ortiz et al., 2015). However, this primarily applies to English due to its structural simplicity (Xue, 2003). Chinese sentences, lacking word separators and context-dependent word meanings, complicate analysis (Ding et al., 2021), posing challenges both in Chinese comprehension and in programming expertise for researchers.

**FIGURE 1:**
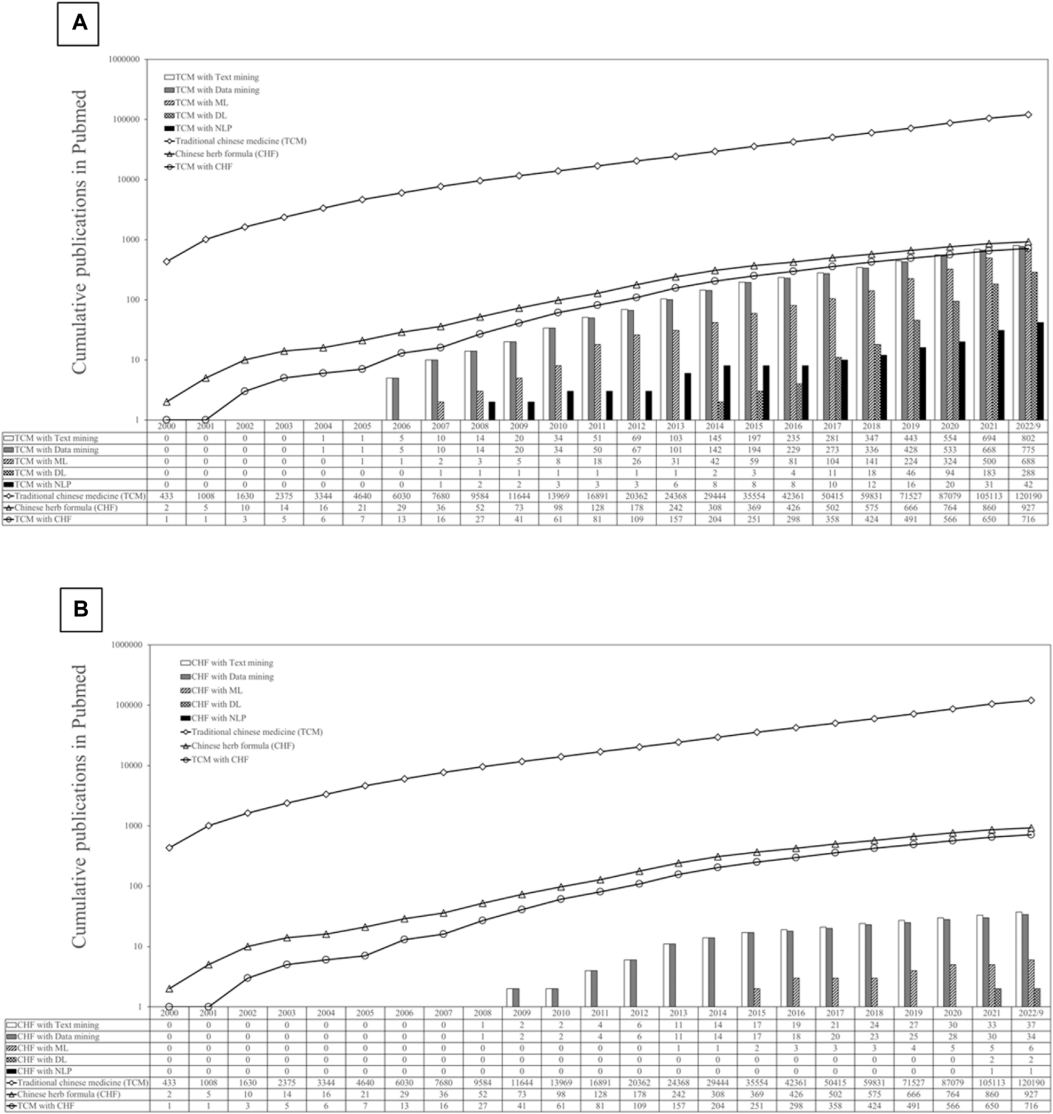
Trends in PubMed indexing of traditional Chinese medicine keywords. **(A)** shows the trend of text mining and NLP in traditional Chinese medicine, while **(B)** depicts the same for Chinese herb formulas (CHF).

Recent years have emphasized the significance of word segmentation, aiding generative language models for diverse applications, including medical services such as electronic record narratives (Lee, 2018; Cong et al., 2019; Zhao et al., 2020; Selivanov et al., 2023). Jieba module simplifies Chinese segmentation (Tsai et al., 2021). Enhancing precision in TCM text segmentation involves supplementing existing tools with TCM corpora (Zhao et al., 2020). We employed PubMed for a keyword cross-search, integrating data science and TCM terms to quantify relevant publications, revealing growth in TCM and Chinese herbal formula research (Figure 1). Amid increased single-herb studies, identifying keywords for formula combinations remains a challenge. Researchers often overlook labor-intensive corpus construction and programming skills needed for TCM text analysis.

We annotated classical texts and herb-pair combinations with 19,328 formula-related and 7,864 herb-related keywords. Association rules identified herb pairs in ancient texts, unveiling new formulas. Chong He et al. noted PubMed’s role in detecting research trends in Chinese medicine (University and Li, 2015). We used herb pairs from Pu-Ji Fang for gene-herb cross-searches on PubMed, confirming correlations through Chi-square or Fisher’s exact tests. A Pu-Ji Fang-based LSTM generative model produced potential herb pairs and formulas. Applying a word count threshold improved the model, supporting diverse herbal portfolio tasks via TCM-LSTM for formula exploration.

## 2 Materials and methods

### 2.1 Data collection and corpus building

We compiled modern Chinese medicine and ancient texts, creating a TCM corpus for analysis. Acquiring the keyword is the main objective. To identify words for extraction in our work about entity nomenclature identification and database building involving ancient Chinese medical texts, we focus on terms related to herbal formulas, ingredients, and their respective pairs. Here are examples of information extracted by the regular expressions:

(a) **Formula Names:** Words that denote specific herbal formulas, such as “Ma Huang Tang” (麻黄汤) or “Liu Wei Di Huang Wan” (六味地黄丸).
(b) **Herbs and Ingredients:** Terms referring to individual herbs or ingredients used in formulas, such as “Ren Shen” (人参, ginseng), “Huang Qi” (黄芪, astragalus), or “Gan Cao” (甘草, licorice root).
(c) **Dosage and Administration:** Words indicating dosage or administration methods, like “份量” (dosage) or “内服” (internal administration).
(d) **Symptoms and Conditions:** Words describing symptoms or conditions targeted by the formulas, such as “頭痛” (headache) or “消化不良” (indigestion).

**FIGURE 2:**
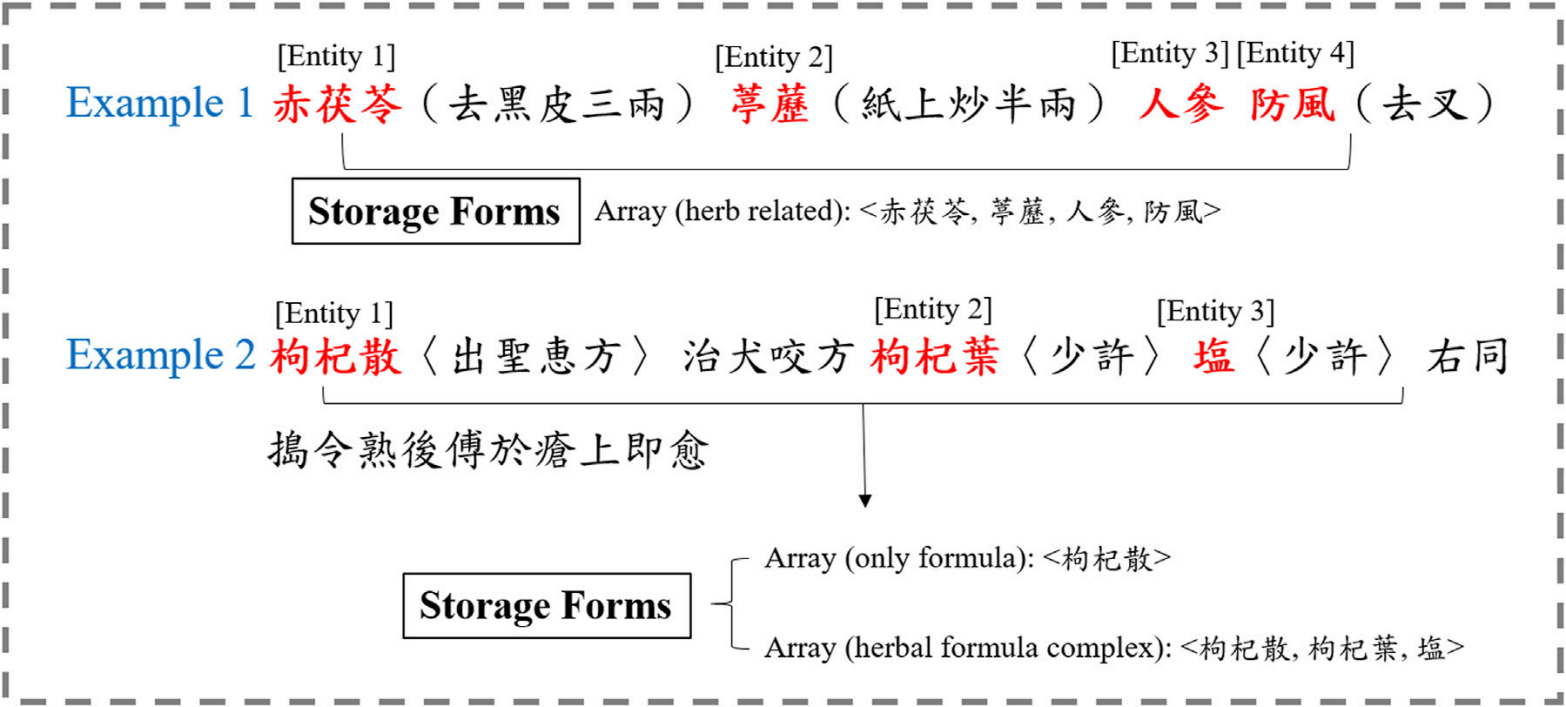
An example of formulas and herbal entities extracted for recognition.

Figure 2 presents an example of the sentences we extracted, demonstrating the practical application of our approach. Table 1 provides an example of the relevant canonical expression used to extract the sentence. The entity is related to herbal combinations and not to genes.

**TABLE 1:** Examples of text keyword extraction in TCM using regular expressions.

Item	Regular expression	Example of sample	Properties of objects extracted	References
1	.*? ()	Example 1. 赤茯苓(去黑皮三兩) 葶藶(紙上炒半兩) 人參 防風(去叉) 澤瀉 甘草(炙銼) 桂(去粗皮) 白朮 野狼毒(銼醋炒) 蜀椒(去目並閉口者炒出汗) 乾薑(炮) 赤小豆(炒各一兩) 大戟(半兩) 肉蓯蓉(酒浸切焙) 豬苓(去黑皮) 女葳(各三分) or Example 2. 丹砂(一兩半研如皂子大絹袋盛以蕎麥灰下汁煮三覆時取出研如粉) or Example 3. 後草藥外,更留鐘乳水三合,磨生犀角三分) 苦參 遠志(去心) 巴戟天(去心)	Herbal name and dosage or method of preparation	Sheng Ji Zong Lu
2	治.*?方	Example 1. 治肝臟中風。筋脈攣急。口眼 斜。言語謇澀。神思昏憒。宜服牛黃散方。 or Example 2. 治頭痛不止。心神煩悶。宜服石膏丸方	Symptom Description	Taiping Sheng Hui Fang
3	.*湯\s治.*	Example 1. 茯苓補心湯	Formula Name and Symptom Description	Bei Ji Qian Jin Yao Fang
		治心氣不足,善悲愁恚怒,衄血,面黃煩悶,五心熱,或獨語不覺,咽喉痛,舌本強,冷涎出(一作汗出)		
		Example 2. 半夏補心湯		
		治心虛寒,心中脹滿悲憂,或夢山丘平澤者方。		
4	治.*用.*	Example 1. 治猪嚙方 用松脂煉作餅子貼上	Symptoms and Usage (in one line)	Pu-Ji Fang 306 vols
5	治.*\s*用.*	Example 1. 治熊傷人瘡	Symptoms and Usage (Text contains line breaks)	Pu-Ji Fang 306 vols
		用蒴藋一大把剉碎以水一升漬須㬰取汁飲餘滓封裹瘡		

We used content catalogs from contemporary e-books, such as the Dictionary of Chinese Medicine, to ensure accurate keyword identification. Ancient Chinese medicine e-books were sourced from Wikipedia, including Pu-Ji Fang (普濟方), Ben Cao Bei Yao (本草備要), Sheng Ji Zong Lu (聖濟總錄), Shi Yi De Xiao Fang (世醫得效方), Taiping Sheng Hui Fang (太平聖惠方), Bei Ji Qian Jin Yao Fang (備急 千金要方), and Zhou Hou Bei Ji Fang (肘後備急方), for text analysis and keyword refinement. Pu-Ji Fang notably integrated early records and was compiled mainly by Zhu Su in the Ming Dynasty, aided by references from healers, various theories, and scriptures (Hou and Jin, 2005; Buck, 2015). Si Ku Quan Shu (四庫全書), created during the Qing Dynasty, contains the reorganized Pu-Ji Fang, offering extensive TCM literature on acupuncture, formulas, vital energy, and more (Lulu, 2022). The contemporary e-book version draws from Qing Dynasty (清朝) information, encompassing diverse medical topics and therapies, preserving pre-15th-century medical knowledge in China.

### 2.2 Extraction of key terms from Chinese medicine texts using regular expressions and manual annotation

We employ regular expressions and manual annotations to access key terms from TCM literature. We applied regular expressions to filter formulas and herb data. Correct matching of various texts with respective patterns renders them herbal keywords. These herbal words, shown in Table 1, are matched using suitable regular expressions. Note that Table 1 contains illustrative terms, not an exhaustive list. Subsequently, formulae and herbs are manually extracted from TCM phrases and cataloged in diverse database tables.

### 2.3 Association rule

The *Apriori* algorithm, a classic in data mining and machine learning (Han et al., 2022), identifies frequent item sets in large datasets, enabling the generation of association rules to depict item relationships. In the *Apriori* algorithm, support refers to the proportion of transactions in the dataset that contain a particular itemset. The formula to calculate support is:

$$\text{Support}\left(X\right)=\frac{\text{number}\;\text{of}\;\text{herbal}\;\text{combination}\;\text{containing}\;X}{\text{total}\;\text{number}\;\text{of}\;\text{herbal}\;\text{combination}}$$

Where:

(a) X represents an itemset.
(b) “Number of herbal combination containing X″ refers to the count of herbal combination in which the itemset X appears.
(c) “Total number of herbal combination” denotes the total count of herbal combination in the dataset.

Support value ranges between 0 and 1, indicating the frequency of occurrence of the itemset X in the dataset. A higher support value signifies that the itemset is more frequent in the dataset.

The *Apriori* algorithm not only identifies frequent herbal entity sets but also derives association rules based on measures like confidence and lift. Confidence quantifies the reliability of an association rule, indicating the likelihood of occurrence of the consequent herbal entity given the antecedent herbal entity(s). It is calculated as the ratio of the support of the combined herbal entity set (antecedent and consequent) to the support of the antecedent herbal entity set alone. Mathematically, confidence is represented as:

$$Confidence\left(X\to Y\right)=\frac{\text{support}\left(X\cup Y\right)}{\text{support}\left(X\right)}$$

where X represents the antecedent herbal entity set, Y denotes the consequent herbal entity, and X→Y represents the association rule.

On the other hand, lift measures the strength of association between two herbal entitys by comparing the observed support of the combined herbal entity set to the expected support if the herbal entitys were independent. It is calculated as:

$$lift\left(X\to Y\right)=\frac{\text{support}\left(X\cup Y\right)}{\text{support}\left(X\right)\times \text{support}\left(Y\right)}$$

A lift value greater than 1 indicates that the presence of the antecedent herbal entity(s) increases the likelihood of occurrence of the consequent herbal entity, suggesting a positive association. A lift value of 1 implies independence between the herbal entitys, while a value less than 1 indicates a negative association.

Both confidence and lift are essential metrics in association rule mining as they help in identifying meaningful and actionable patterns in the data. High confidence and lift values signify strong associations between herbal entitys, making them valuable for decision-making processes such as herbs recommendations in formulas and herbal candidate for substitution rare substance.

The *Apriori* property and support measures boosted algorithm efficiency. The low frequent item in dataset implies that infrequent itemsets and subsets share rarity, curtailing non-frequent subset analysis. The support measure gauges frequency via transaction proportion. Our iterative approach identifies frequent keyword item sets from TCM text, progressing from surpassing a threshold to pairs, triples, and larger sets.

We utilize the Python library mlxtend 0.19.0 (Machine Learning Extension) to execute the *Apriori* algorithm. This discovery process employs the *Apriori* algorithm to identify potential TCM prescription combinations and relationships within data.

### 2.4 Gene set preparation

We selected multiple sets of genes we were interested in from the KEGG human disease pathway (Kanehisa et al., 2023), which are cardiovascular disease (n = 583), specific types of cancer pathway (n = 479), and respiratory disease pathway (n = 139). The respiratory pathway consists of the asthma and lung cancer cell pathways.

### 2.5 Potential herbal pair exploration

To determine currently the English or Latin names of the herbs studied from ancient texts, we referred to 703 herb names provided by the SymMap database (Wu et al., 2019). We extracted herbal keywords from Pu-Ji Fang and intersected them with the SymMap Herbal name list as the potential herb pairs candidates.

### 2.6 Exploring the impact of commonly used herbs on medicine, pharmacology, and traditional Chinese medicine: a case study

Chinese Angelica, known as Dang Gui in Chinese, is often paired with licorice (Yang et al., 2015). With advances in research techniques, more and more studies are examining preparations combining multiple herbs. For example, medicinal prescriptions contain chamomile, silverweed, licorice, angelica, blessed thistle, and wormwood for alleviating gastrointestinal disorders (Wegener and Heimueller, 2016). Moreover, studies on the extraction of single herbal compounds for disease treatment, such as licorice extracts for liver disease (Li et al., 2014), suggest that utilizing TCM formulas and single herbs is gaining prominence. In medicine and pharmacology, data mining techniques extract valuable insights from large datasets, identifying patterns, correlations, and associations among variables (Chu et al., 2020; Wu et al., 2021). A notable example is herb-herb networks, which elucidate the mechanism of herb pairs based on composition and targeting, determining the significance of synergistic effects in disease treatment (Wang et al., 2021).

We extracted keywords from classical Chinese medical literature and analyzed association rules between them using the *Apriori* algorithm. Our aimed to identify relationships between entities and comprehend the prescribing conventions in ancient languages. Additionally, the above references suggest that multiple herbs synergize with a central formula. To explore the potential mechanism of this central component, we created a network diagram of keyword entities from ancient texts. The network helped us understand spatial distances between entities within formulae and herb pairs.

Furthermore, studying the mechanism of action of herbal formulations in the human body is challenging due to the complexity of herbal mixtures, comprising numerous compounds targeting multiple cellular sites (Chen et al., 2016). Additionally, analyzing modern research on the relationship between herbs and genes is essential (Fang et al., 2008), considering the historical context of ancient texts (Chang, 2016). Here, we obtained gene sets related to heart, lung, and cancer from KEGG as gene references. Pu-Ji Fang is well-classified in disease classification for Chinese herbs. Thus, we utilized ancient keywords of the five viscera and five bowels (excluding San Jiao “三焦”) to identify co-occurring herbs. Subsequently, we utilized these herbal entities to conduct a PubMed search using pooled gene entities. Commonly used herbs licorice (Yang et al., 2015) served as control and widely-used anticancer TCM drugs Shenqifuzheng Injection served as the positive control group for Chi-square testing. The objective is to comprehend the investigation of co-occurring herbs in classical TCM texts and the genome within contemporary scientific research. Next, we retrieved the union results of herb entities and gene keywords (references counts) from PubMed. Subsequently, the number of references for licorice, a commonly used Chinese medicine, was employed as the control group, while the number of references for the widely-used anticancer drug Shenqifuzheng Injection (Wu et al., 2015) served as the positive control group. The literature counts under the three conditions were then subjected to a Chi-square test to elucidate contemporary scientific research on co-occurring herbs and genomes.

### 2.7 LSTM-based formulation of traditional Chinese medicine recipes

We employed the PyTorch framework in a Windows 10 server with 24 GB RAM for our pipeline. We trained the model with an NVIDIA GTX 1050 GPU with 2 GB RAM. The aim is to create a generative model capturing herb-pair distribution patterns in historical Chinese medical texts, generating new instances based on learned distributions. Deep learning and generative modeling reveal interdependencies between formulations and botanical constituents in ancient Chinese medical keywords. Increasing epochs deepens model knowledge, aligning sample distribution with classical text patterns. We reference (Joshi, 2024) and adapt it for our herbal keyword assemblage. Our approach centers on the Long Short-Term Memory (LSTM) architecture (Hochreiter and Schmidhuber, 1997) for generating herbal recipes by responding to inputs of formulas and herbs.

### 2.8 Statistics

We conducted PubMed cross-searches using Pu-Ji Fang keywords and KEGG gene sets. Outcomes formed 3 × 2 contingency tables, using chi-square for samples >30 and Fisher’s exact test for <30. The control group comprised high-frequency herbs from the Pu-Ji Fang Classification. The positive control was Shenqifuzheng Injection, a widely-used cancer treatment (Wu et al., 2015). The aim is to delineate two conditions: (1) Whether discrepancies exist in the volume of extant literature for distinct genes within the same herb. (2) Whether variations are apparent in the quantity of existing literature for different herbs targeting the same gene. Due to the relevance of the Ulcer-related Phylum (癰疽門) in Pu-Ji Fang, encompassing characteristics of abscesses and tumors, we opted for Shenqifuzheng Injection entities as the positive control group for cross-searching literature counts. The Python module Scipy and RPY2 package (https://rpy.sourceforge.io/rpy2.html) facilitated Chi-square and Fisher’s exact tests, enhancing the findings’ reliability and significance.

## 3 Results

### 3.1 Enhanced text analysis and keyword retrieval for traditional Chinese medicine research using color-coded labels and iterative approaches

Contemporary systems biology aligns with the holistic approach of Chinese medicine, in contrast to Western reductionism (Yan et al., 2014). However, the intricate herbal formulas of Chinese medicine pose challenges for global herbal research. Historical insights guide the pairing of herbs, necessitating keyword recognition, and iterative optimization.

We employed regular expressions to extract keywords, forming a database from classical texts. Formula keywords are inherently complex due to various terms and combinations (School of Chinese Medicine, 2014). Our workflow involves categorization, manual identification, and dataset refinement (Supplemental data is available upon request). Additionally, we manually added unidentified texts containing keywords, effectively resolving issues with phrase recognition.

**FIGURE 3:**
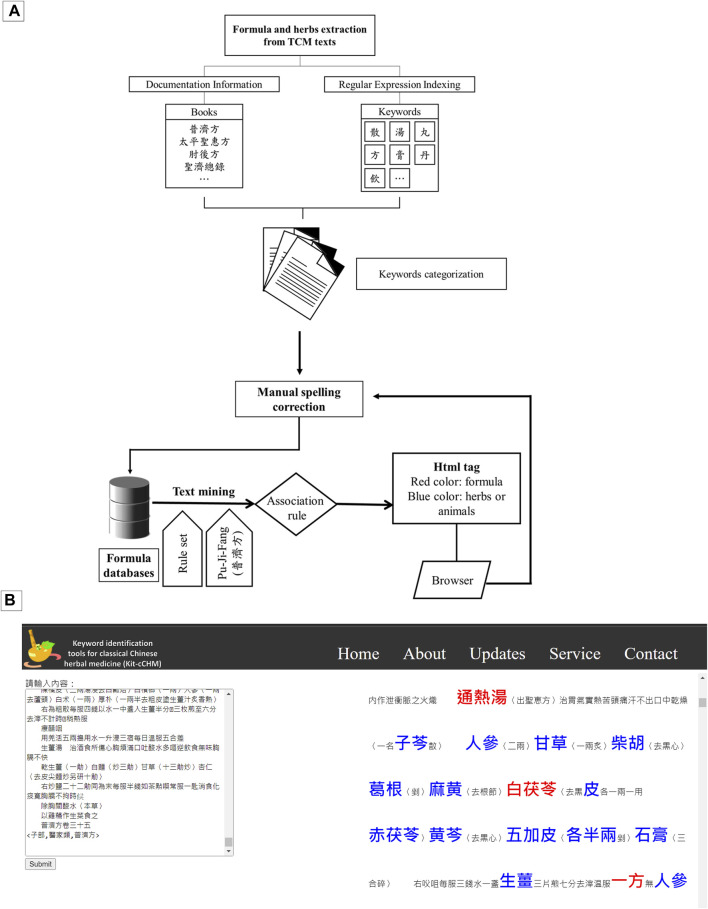
Illustration of data collection process and visualization schematics. **(A)** Flowchart illustrating the process of keyword extraction from ancient texts (source from Wikipedia e-book and mentioned in Methods 2.1); **(B)** Presentation of the keyword extraction results, with examples taken from the contents of Pu-Ji Fang.

**FIGURE 4:**
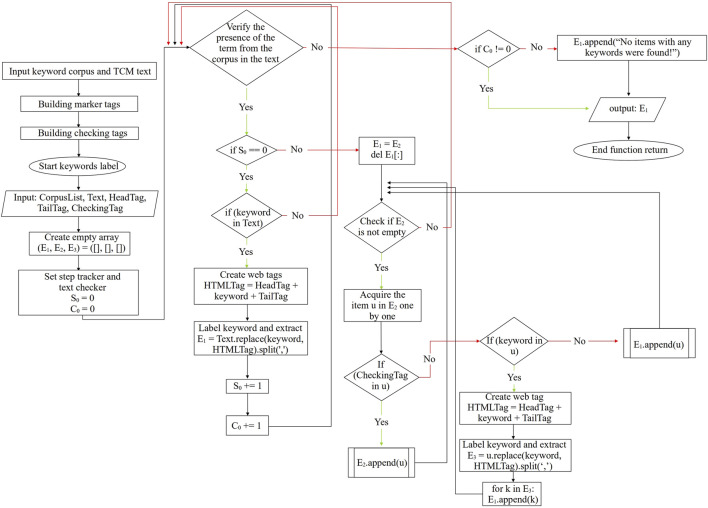
Demonstration of iterative algorithms for formulas and herbal extracts.

Figure 3A outlines our text processing workflow, while users distinguish between formula and herb keywords through color-coded labels in web browsers (Figure 3B). Figure 4 illustrates the tagging process for ancient Chinese medicine texts using web forms for text analysis submission. We marked formulas with red tags and designated herbs with blue labels. Chinese word segmentation differs from using spaces in English, and Chinese word breaks vary from those in English. We preserve meaning and use commas to separate keywords to account for diverse character encodings. Sorting keywords by length prevents re-segmentation during tagging, facilitating extensive keyword retrieval. This iterative approach aids in identifying Chinese medicine products, ultimately reducing the labor intensity associated with Traditional Chinese Medicine (TCM) analysis.

### 3.2 Exploration of ancient Chinese medical texts for potential pharmaceutical research via keyword extraction, data processing and LSTM language generation

**FIGURE 5:**
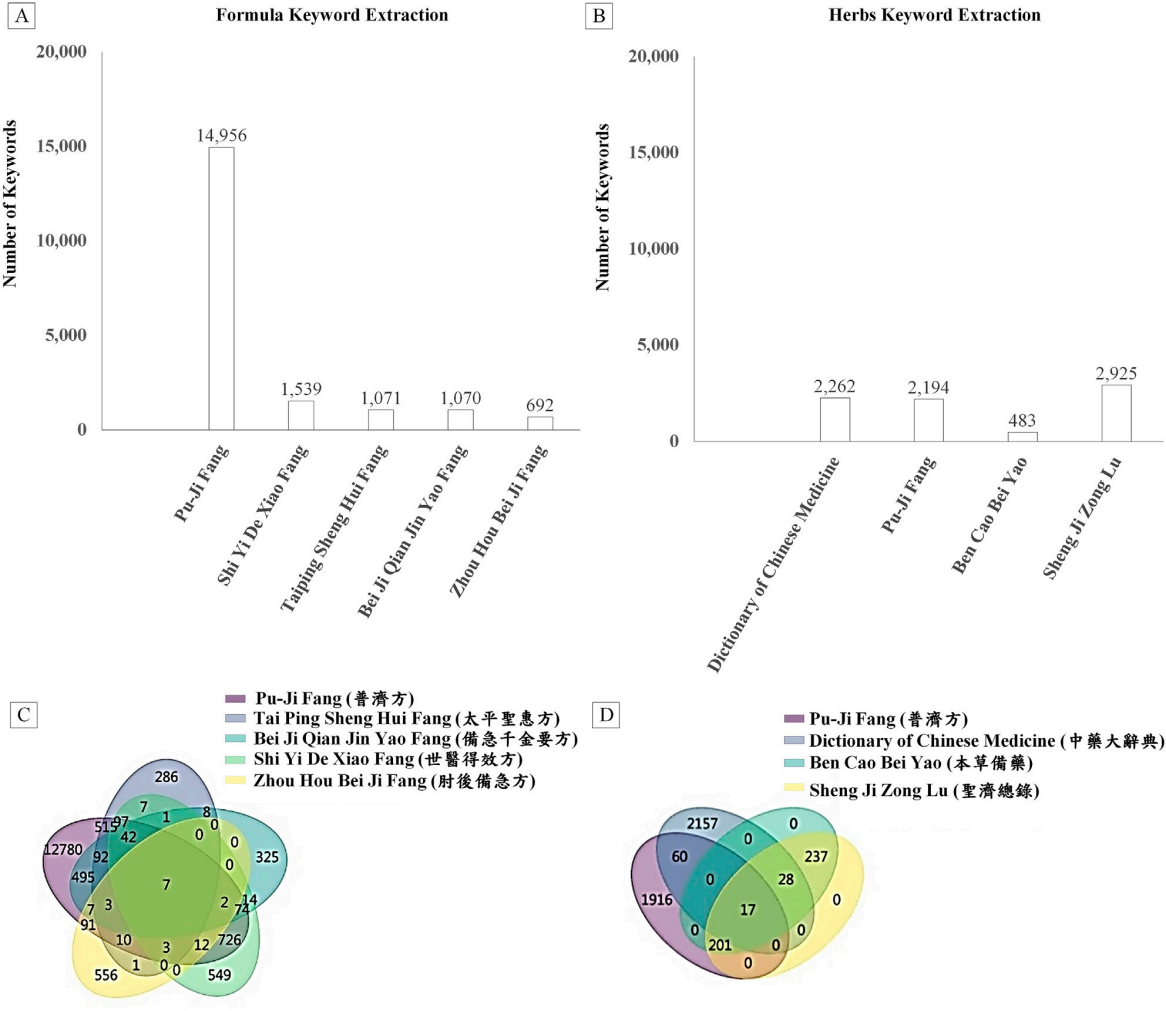
Histograms and union charts showing the number of keywords from various TCM texts. **(A)** Histogram depicting keyword counts in formulas; **(B)** Histogram illustrating keyword counts in herbs; **(C)** Union charts representing formula occurrences across various TCM texts; **(D)** Union charts displaying herb occurrences across various TCM texts.

Figure 5A illustrates the counts of extracted formula keywords: Pu-Ji Fang (14,956), Shi Yi De Xiao Fang (1,539), Taiping Sheng Hui Fang (1,071), Bei Ji Qian Jin Yao Fang (1,070), and Zhou Hou Bei Ji Fang (692). In Figure 5B, we presented the herb keyword counts: Pu-Ji Fang (2,262), Ben Cao Bei Yao (2,194), Sheng Ji Zong Lu (483), and Dictionary of Chinese Medicine (2,925).

To prevent our program from tagging the same keyword repeatedly during the iterative labeling process, we employed a union approach to exclude duplicates (Figures 5C,D). Subsequently, we stored these data in the database. Moreover, some terms refer to restricted toxic herbs or prohibited rare animals and plants. We do not endorse their clinical use or consumption.

**FIGURE 6:**
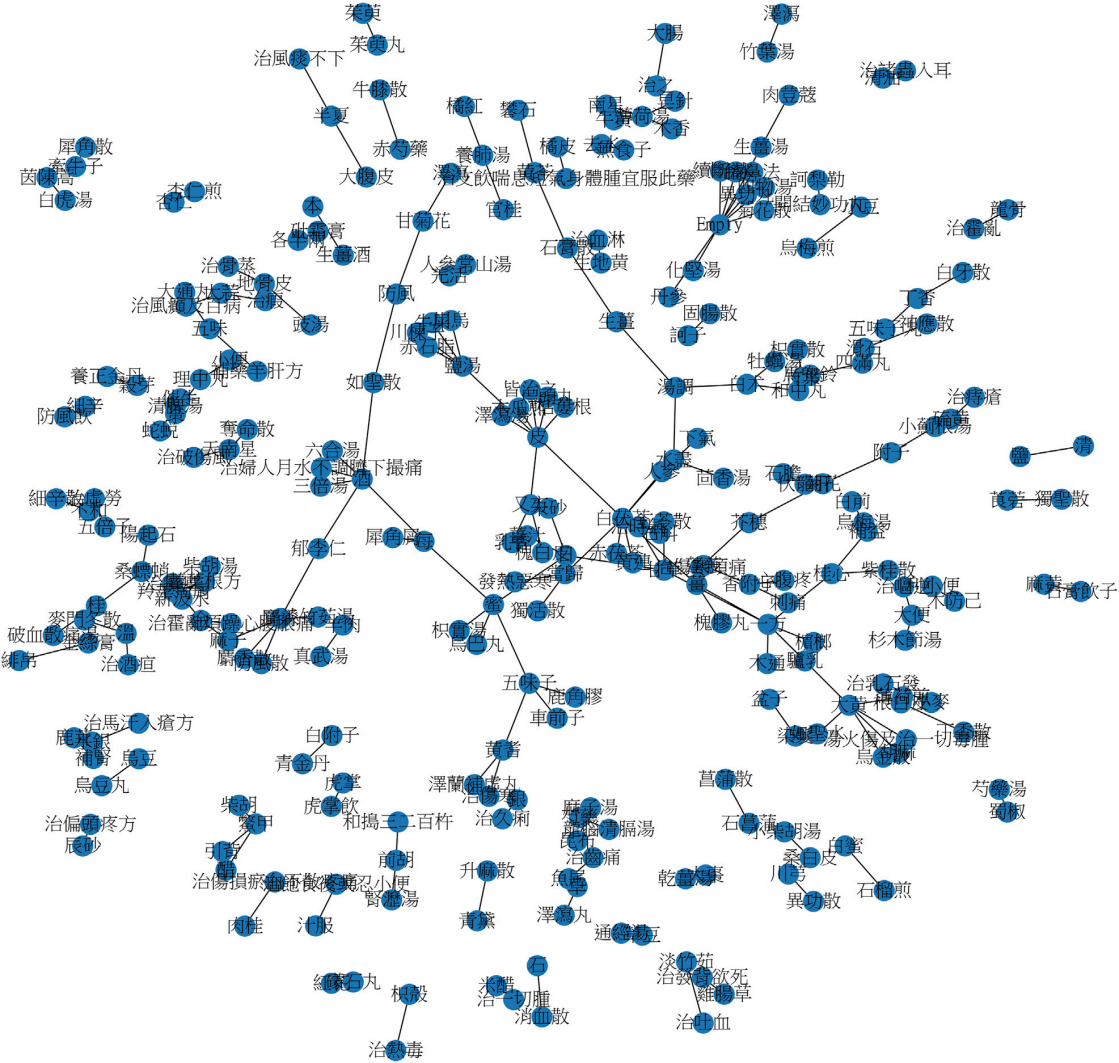
Network of keywords associated with Chinese herbal pair combinations.

The endeavor of deriving potential new drugs from Chinese medicinal texts is a labor-intensive task. Classical TCM literature documents the therapeutic processes underlying human diseases, searching for novel drug combinations complex. Additionally, training personnel to interpret ancient narratives incurs substantial expenses, impeding systematic research. Therefore, we primarily focus on extracting formulas and herb pairs from Pu-Ji Fang. We amassed a collection of 16,384 keyword combinations from its 426 volumes and subsequently performed a random selection of 200 herb pairs. We showed the resulting network in Figure 6, where nodes represent formulas/herbs and segments denote herb counts. More extensive investigation involves incorporating this network with other networks, including genetic, proteomic, disease, and drug networks.

**FIGURE 7:**
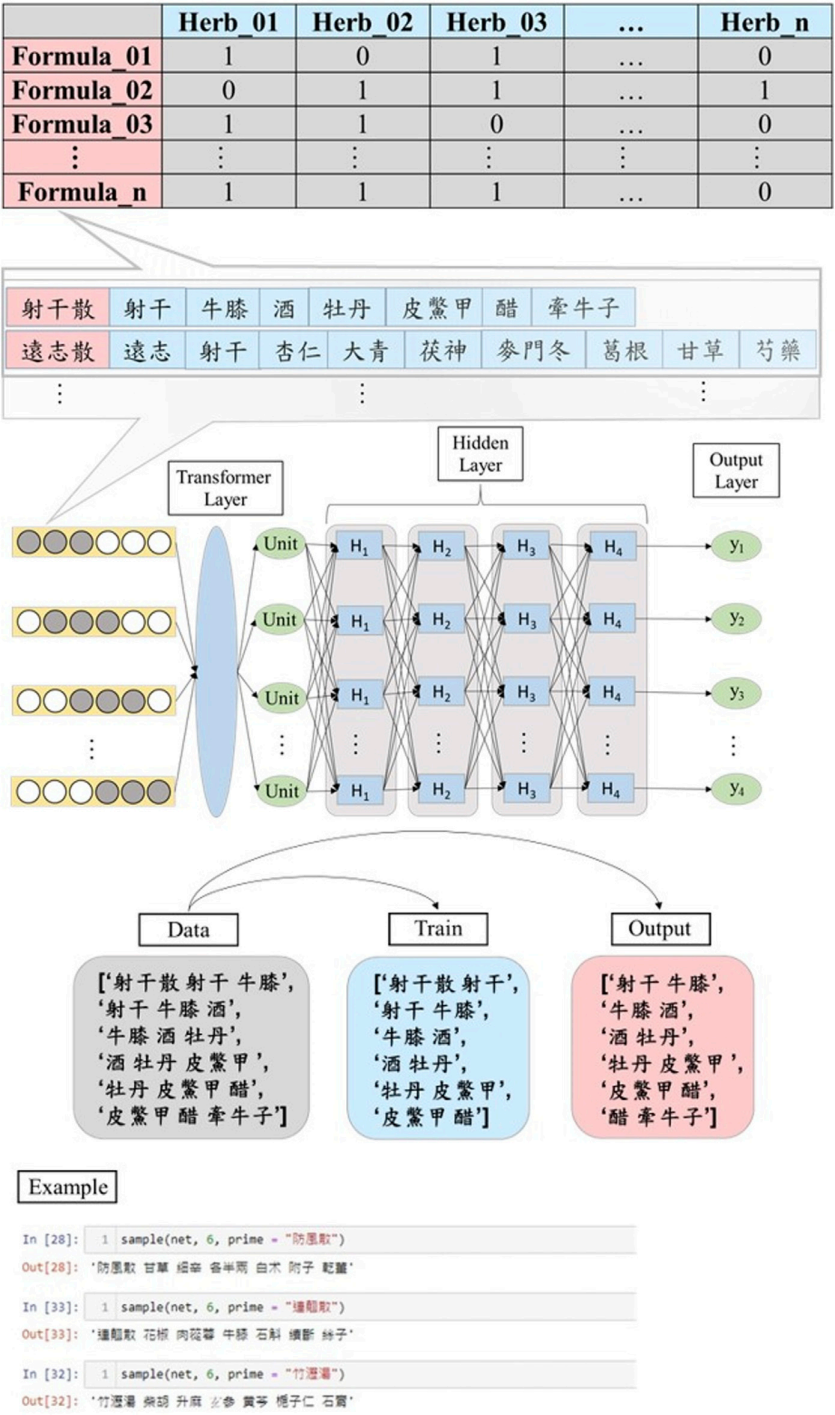
LSTM generates formulas via keyword extraction from classical textual data.

Recent years saw ML and NLP advancements, offering promise across domains, including pharmaceutical data mining. Chinese Medicine Electronic Medical Records (CEMR) hold untapped potential, hindered by heterogeneity and privacy concerns. Accessing EMRs is challenging, curbing ML and NLP progress. Figure 7 presents our LSTM-based ancient Chinese medicine text model, training on Pu-Ji Fang’s keywords. This model generates synthetic narratives, aiding research without compromising confidentiality. This approach supports downstream ML and NLP, enhancing understanding of Traditional Chinese Medicine’s therapeutic potential in global healthcare.

### 3.3 association rule analysis of keyword itemsets for Chinese Medicine text extraction based on Apriori algorithm

Traditional Chinese Medicine (TCM) divides prescriptions into four categories: monarch (main) drugs (君藥), minister drugs (臣藥), assistant drugs (佐藥), and guide drugs (使藥) (Wang et al., 2013). These categories pertain to rules regarding the use of prescriptions and the relationships between primary and secondary effects, guiding the usage of formulas in TCM. Monarch drugs have a central therapeutic effect for treating the primary ailment. Minister drugs target symptoms and the primary disease extension. Assisted drugs alleviate adverse effects and improve therapeutic benefits. Guide drugs enhance the effects of other ingredients, lessen toxicity, or improve the taste (Wang et al., 2013; Yongxiang, 2015).

Although we do not have information about which herbs belong to which categories, we expect that there are frequent relationships between herbs. According to the results obtained from the *Apriori* algorithm, we found several interesting herbs listed in paragraph.

Liquorice, or Gan Ciao (甘草) in Chinese, the root of Glycyrrhiza uralensis Fisch or Glycyrrhiza glabra Leguminosae, is native to Europe and Asia and has been used as a medicine and food. Traditional Chinese medicine frequently uses it in combination with other herbs as a guide drug to enhance the effectiveness of other ingredients, lessen toxicity, and improve palatability (Wang et al., 2013).

Dang Gui (當歸), the root of a perennial herb belonging to the Apiaceae family, is extensively utilized in Chinese herbal medicine (Wu and Hsieh, 2011). Dang Gui is a crucial component in Si Wu (四物), one of the most well-known herbal formulas. In traditional Chinese medicine, Dang Gui is classified as a minister drug and is commonly employed to promote blood circulation and regulate the immune system (Du et al., 2020).

ZhiQiao (枳殼), the dried unripe fruit of Citrus aurantium L., is of significant interest due to its high content of phenolic compounds with health-promoting effects (Zhang and Feng, 2019; Ding et al., 2020). Worldwide, citrus fruits and juices are popular due to their high nutritional content and delicious flavor (Zhao et al., 2017).

Honey, or “蜜” in Chinese, holds a significant position in traditional Chinese medicine and is commonly utilized as an additive to single herbal remedies, chemicals, or other forms of medications as an adjuvant, including formulations like honey pills (蜜丸). The major component of honey has similar molar ratios to glucose and fructose, which makes honey a natural deep eutectic solvent (NADES). NADES are known to be exceptional solvents for moderately polar bioactive compounds and can be utilized in pharmaceutical formulations to enhance the efficacy of herbs and stabilize active compounds (Dai et al., 2021).

Our corpus identifies the essential herb terms and correlates them with current knowledge. Further information produced by the *Apriori* algorithm is obtainable from the Supplementary Data. Using a TCM keyword iterative approach, we analyzed the association rules of the whole Pu-Ji Fang. By calculating the frequency of herbal keywords using the *Apriori* algorithm with a 0.8 support threshold, the results indicated lift >1, indicating a positive correlation between keyword pairs in the formulas. After conducting the association rule analysis, Table 2 show the frequencies of keyword occurrences. We assessed the support of the herbal item set by examining the proportion of sub-items contained within the formula item set. As depicted in Table 2, the combination of licorice and honey exhibits a support value of 0.93, signifying that 93% of this pairing is present within our current dataset. Nevertheless, residual noise persists in our analyses despite the steps taken to clear the data of non-herbal keywords. This challenge underscores the need for future manual inspection efforts. The combination of Angelica and licorice, recognized as herbal constituents, exhibits a support value of 0.91 in our dataset, suggesting that 91% of our current dataset features this pairing. The confidence value denotes the likelihood of selecting herb Y when herb X is chosen, symbolized as {X → Y}. For the co-occurrence of Angelica and licorice, the confidence level stands at 0.992, suggesting a probability of 99.2%. This finding supports the assertion that licorice and Angelica frequently co-occur in conjunction (Yang et al., 2015). The co-occurrence of these two herbs yielded similar outcomes as depicted in Table 3. We rank the degree of lift that suggests a positive correlation among combinations (lift >1), indicating the co-occurrence of either Angelica and licorice or alongside vinegar, honey, and ZhiQiao. Currently, our results require the support of evidence from available information on the profile of Chinese herbal medicines with relevant references. Vinegar and honey serve as adjuncts in the processing of Chinese medicines, as reported in the review paper authored by Lin-Lin Chen et al. (Chen et al., 2018). Existing reference delves into studies concerning metabolic syndromes that incorporate licorice, ZhiQiao, and other herbs, with ZhiQiao identified as the third significant herb in treating metabolic syndrome in that particular order; this corresponds with the delineation of the four categories in various prescriptions as aforementioned (Chen et al., 2016). Moreover, the varying effects of herb pairs in the formulations on ZhiQiao necessitate further investigation in future studies. Current findings underscore the need for continued investigation to ascertain the specific agents utilized. Our research is warranted to elucidate the exact formulations employed in light of the diverse effects observed among herb pairs in the future. It is worth noting that most keywords lack prescription names, suggesting that herbs and animals could be used for taste rather than for treatment purposes in the formulas. We consider it possible that ancient healers employed them to enhance the flavor rather than for therapeutic benefits. Several studies support the interpretation that some ingredients do not always contain the prescription, indicating that they are not directly related to the treatment (Wang et al., 2013; Yongxiang, 2015; Du et al., 2020). Overall, this analysis provides insight into the potential role of these herbs or animals in TCM prescriptions.

**TABLE 2:** Application of the *Apriori* algorithm to analyze herbal relationships in the Pu-Ji Fang traditional Chinese medicine literature. Application of Apriori algorithm in Chinese medicine data analysis in descending order of support.

Antecedents	Consequents	Antecedent support	Consequent support	Support	Confidence	Lift	Leverage	Conviction
['蜜']	['甘草']	0.939	0.947	0.930	0.990	1.045	0.040	5.167
['甘草']	['蜜']	0.947	0.939	0.930	0.982	1.045	0.040	3.381
['清']	['甘草']	0.978	0.947	0.927	0.948	1.001	0.001	1.025
['甘草']	['清']	0.947	0.978	0.927	0.980	1.001	0.001	1.065
['清']	['蜜']	0.978	0.939	0.920	0.941	1.001	0.001	1.019
['蜜']	['清']	0.939	0.978	0.920	0.979	1.001	0.001	1.057
['清']	['木']	0.978	0.932	0.915	0.936	1.004	0.003	1.053
['木']	['清']	0.932	0.978	0.915	0.982	1.004	0.003	1.199
['甘草']	['蜜', '清']	0.947	0.920	0.910	0.962	1.045	0.039	2.083
['醋']	['蜜']	0.927	0.939	0.910	0.982	1.045	0.039	3.312
['當歸']	['甘草']	0.918	0.947	0.910	0.992	1.048	0.042	6.730
['醋']	['甘草']	0.927	0.947	0.910	0.982	1.037	0.032	2.915
['甘草']	['當歸']	0.947	0.918	0.910	0.962	1.048	0.042	2.146
['蜜']	['醋']	0.939	0.927	0.910	0.969	1.045	0.039	2.349
['蜜']	['清', '甘草']	0.939	0.927	0.910	0.969	1.045	0.039	2.349
['清', '甘草']	['蜜']	0.927	0.939	0.910	0.982	1.045	0.039	3.312
['蜜', '清']	['甘草']	0.920	0.947	0.910	0.989	1.045	0.039	5.061
['清']	['蜜', '甘草']	0.978	0.930	0.910	0.931	1.001	0.001	1.013
['蜜', '甘草']	['清']	0.930	0.978	0.910	0.979	1.001	0.001	1.046
['甘草']	['醋']	0.947	0.927	0.910	0.962	1.037	0.032	1.893

**TABLE 3:** Application of Apriori algorithm in Chinese medicine data analysis in descending order of lift.

Antecedents	Consequents	Antecedent support	Consequent support	Support	Confidence	Lift	Leverage	Conviction
['當歸', '醋', '甘草']	['枳殻', '蜜']	0.886	0.821	0.804	0.907	1.105	0.076	1.929
['枳殻', '蜜']	['當歸', '醋', '甘草']	0.821	0.886	0.804	0.979	1.105	0.076	5.511
['當歸', '蜜', '醋', '甘草']	['枳殻']	0.884	0.823	0.804	0.910	1.105	0.076	1.955
['枳殻']	['當歸', '蜜', '醋', '甘草']	0.823	0.884	0.804	0.976	1.105	0.076	4.939
['大黄']	['黄芩']	0.852	0.852	0.801	0.940	1.103	0.075	2.476
['黄芩']	['大黄']	0.852	0.852	0.801	0.940	1.103	0.075	2.476
['當歸', '醋']	['枳殻', '蜜']	0.891	0.821	0.806	0.905	1.102	0.075	1.884
['枳殻', '蜜']	['當歸', '醋']	0.821	0.891	0.806	0.982	1.102	0.075	6.156
['枳殻', '蜜', '甘草']	['當歸', '醋']	0.818	0.891	0.804	0.982	1.102	0.075	6.138
['當歸', '醋']	['枳殻', '蜜', '甘草']	0.891	0.818	0.804	0.902	1.102	0.075	1.856
['枳殻']	['當歸', '蜜', '醋']	0.823	0.889	0.806	0.979	1.102	0.075	5.410
['當歸', '蜜', '醋']	['枳殻']	0.889	0.823	0.806	0.907	1.102	0.075	1.908
['枳殻', '甘草']	['當歸', '蜜', '醋']	0.821	0.889	0.804	0.979	1.102	0.074	5.394
['當歸', '蜜', '醋']	['枳殻', '甘草']	0.889	0.821	0.804	0.905	1.102	0.074	1.879
['當歸', '醋', '甘草']	['枳殻']	0.886	0.823	0.804	0.907	1.102	0.074	1.903
['枳殻']	['當歸', '醋', '甘草']	0.823	0.886	0.804	0.976	1.102	0.074	4.837
['木香', '當歸']	['白术', '蜜', '醋']	0.872	0.835	0.801	0.919	1.101	0.073	2.044
['白术', '蜜', '醋']	['木香', '當歸']	0.835	0.872	0.801	0.959	1.101	0.073	3.162
['白术', '醋']	['木香', '當歸', '蜜']	0.840	0.867	0.801	0.954	1.100	0.073	2.888
['木香', '當歸', '蜜']	['白术', '醋']	0.867	0.840	0.801	0.925	1.100	0.073	2.119

### 3.4 Frequency of co-occurring herbs in Pu-Ji Fang disease classification, and their relationship to chi-square tests

Ancient medics divided Pu-Ji Fang’s disease topics into 77 phyla based on Chinese medical theories. Using an iterative method, we extracted relevant keywords from these phyla. Liquorice and angelica frequently appeared in 70 disease themes. Following the Five Element Theory, we focused on the heart-related phylum (心臟門) due to its central role in TCM as a vital element of circulation (Chung et al., 2017). Our research covers various organ phyla, including the large intestine (大腸腑門), liver (肝臟門), lung (肺臟門), and more (see Table 4 for details), for comprehensive analysis. In-depth understanding of disease patterns and herb pairs in “Pu-Ji Fang.”

**TABLE 4:** Frequency of co-occurring herbs in Pu-Ji Fang disease classification, chi-square test results.

		Herbs							
		Ginseng (人參)	Largetrifoliolious Bugbane Rhizome (升麻)	Immature fruit of Seville orange (枳實)	Root of Twotooth Achyranthes (牛膝)	Liquorice (甘草)	Angelica (當歸)	Rhizome of common Amarrhe (知母)	Monkshood (附子)
Phylum	Large intestine (大腸腑門)	27	13	23	7	83	49	1	33
	Small intestine (小腸腑門)	1	3	2	4	8	9	1	6
	Heart (心臟門)	103	20	5	6	103	39	12	22
	Ulcer-related (癰疽門)	76	74	15	13	223	149	39	53
	Liver (肝臟門)	3	13	18	28	56	43	3	30
	Lung (肺臟門)	76	18	9	3	110	12	16	22
	Stomach (胃腑門)	61	5	3	1	62	17	3	29
	Spleen (脾臟門)	141	27	40	9	208	72	11	98
	Kidney (腎臟門)	50	4	10	59	59	57	2	86
	Bladder (膀胱腑門)	6	2	2	4	9	5	2	10
	Gallbladder (膽腑門)	24	5	2	1	18	1	4	1

Statistic: 695.2055226775681.

*p*-value: 1.0215460055739217e-103.

Degree of freedom: 70.

*p*-value <0.001: True.

We identified the total number of eight co-occurring herbs. Independent chi-square tests (Table 5) show a significant influence of co-occurring herb combinations on disease categorization as per classical TCM theory.

**TABLE 5:** Determination of candidate herbs through chi-square or Fisher’s exact cross-search.

Herbs	Gene_1	Gene_2	*p*-value	Chi-square	Fisher’s exact test	Significant
Rhizoma Anemarrhee(知母)	CASP3	BCL2L1	0.002688281	T	F	**
Radix Ginseng(人參)	AKT2	STAT3	0.005952393	F	T	**
Rhizoma Anemarrhee(知母)	PTK2	CASP3	0.007672606	F	T	**
Radix Ginseng(人參)	STAT3	BCL2	0.008284285	F	T	**
Rhizoma Anemarrhee(知母)	AKT2	PTK2	0.008846575	F	T	**
Rhizoma Anemarrhee(知母)	PTK2	BAX	0.012105505	F	T	*
Radix Ginseng(人參)	STAT3	BAX	0.012278138	F	T	*
Fructus Aurantii Immaturus(枳實)	CASP3	PRKCG	0.014492754	F	T	*
Aurantii Fructus Immaturus(枳實)	CASP3	PRKCG	0.014492754	F	T	*
Fructus Aurantii Immaturus(枳實)	STAT3	PRKCG	0.015151515	F	T	*
Aurantii Fructus Immaturus(枳實)	STAT3	PRKCG	0.015151515	F	T	*
Rhizoma Anemarrhee(知母)	CASP3	PRKCA	0.016023511	F	T	*
Rhizoma Anemarrhee(知母)	AKT2	PRKCA	0.017236586	F	T	*
Angelicae Sinensis Radix(當歸)	AKT1	KRAS	0.017285862	F	T	*
Radix Angelicae Sinensis(當歸)	AKT1	KRAS	0.017285862	F	T	*
Fructus Aurantii Immaturus(枳實)	PRKCG	CASP9	0.018181818	F	T	*
Aurantii Fructus Immaturus(枳實)	PRKCG	CASP9	0.018181818	F	T	*
Rhizoma Anemarrhee(知母)	PRKCA	BAX	0.020718864	F	T	*
Radix Ginseng(人參)	STAT3	CASP3	0.023239814	F	T	*
Rhizoma Anemarrhee(知母)	PTK2	PRKCB	0.024509804	F	T	*
Rhizoma Anemarrhee(知母)	TP53	AKT2	0.025156031	F	T	*
Rhizoma Anemarrhee(知母)	PTK2	AKT1	0.026075722	F	T	*
Rhizoma Anemarrhee(知母)	TP53	CASP3	0.026353954	T	F	*
Rhizoma Anemarrhee(知母)	AKT1	PTEN	0.026730056	F	T	*
Rhizoma Anemarrhee(知母)	PTK2	CASP9	0.026814558	F	T	*
Radix Ginseng(人參)	STAT3	BCL2L1	0.027634131	F	T	*
Rhizoma Anemarrhee(知母)	PRKCA	PRKCB	0.028571429	F	T	*
Rhizoma Anemarrhee(知母)	PTK2	NFKB1	0.029411765	F	T	*
Rhizoma Anemarrhee(知母)	PTK2	MAPK12	0.031225296	F	T	*

### 3.5 Analyzing the cross-search of genes and herbs in PubMed through KEGG gene sets and Pu-Ji Fang herb-related keywords

**FIGURE 8:**
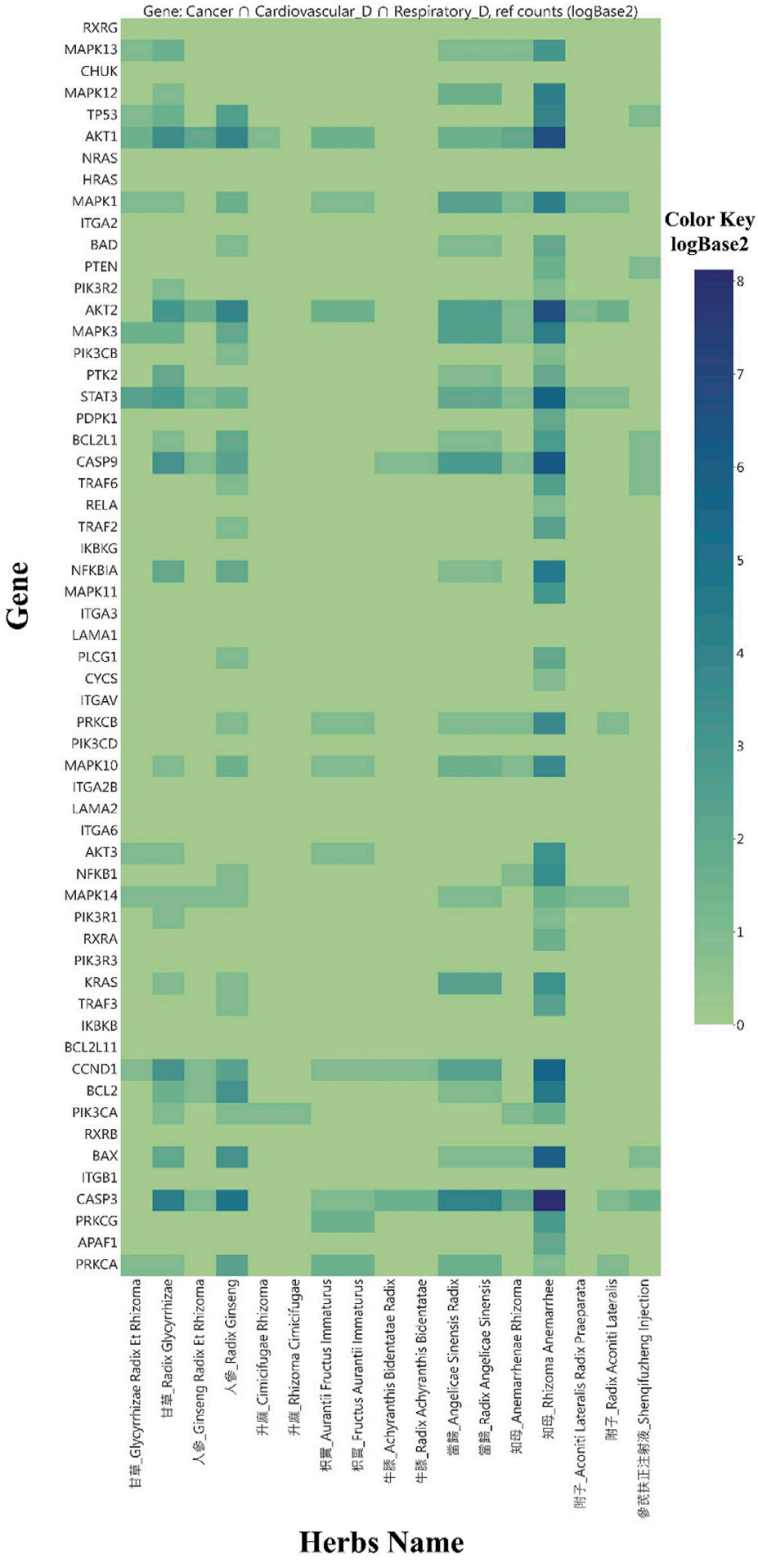
Log2-transformed literature count heatmap for genetic-herb cross-search results.

We conducted Chi-square or Fisher’s exact tests to examine gene-herb associations via co-occurrence in scientific literature, specifically focusing on interactions between target genes and herbs from the Pu-Ji Fang corpus. We gathered 7,664 PubMed cross-search entries for gene-herb associations and 934 entries for Shenqifuzheng Injection, serving as a positive control. A heat map (Figure 8) visually displays gene-herb distribution in modern Chinese medicine research using log2-transformed document counts. We combined all the types of licorice and applied Chi-square/Fisher’s exact tests to herbs and genes, including Shenqifuzheng Injection (Table 5).

## 4 Discussion

### 4.1 The rise of Traditional Chinese Medicine (TCM) in Western healthcare

The acceptance of prescriptions in Western countries reflects a dynamic interplay of various socio-cultural, economic, and healthcare factors (Dobos et al., 2005). Historically, Western medical practices predominantly emphasized pharmaceutical interventions, often overlooking holistic traditional prescriptions (Ma et al., 2021).

Since the 1990s, there has been a burgeoning interest in traditional Chinese medicine (TCM) formulations in Western countries, driven by the quest for alternative and complementary healthcare options (Pearson and Chesney, 2007; Xu et al., 2013). Initially, the government of China emphasized promoting traditional Chinese medicine, leading to the establishment of the State Administration of Traditional Chinese Medicine (SATCM) (Chan, 2005). The institution aims to coordinate matters related to Chinese medicine in China and to promote its practice abroad. Concrete research efforts, such as Chinese medicine integrating with Western medical practices, have played a pivotal role in advancing this trend (Xu et al., 2013). By the 1990s, traditional Chinese medicine had solidified a significant presence in Western countries, marked by the establishment of specialized Chinese medicine hospitals, outpatient facilities, and a cadre of practitioners serving diverse communities (Fu et al., 2021). One of the most significant examples is the first university-based TCM hospital in Germany in 1991; this was a pivotal moment in accepting TCM in Western countries (Melchart et al., 1999). This event marked a remarkable milestone in the expansion of TCM into Western medical practice and the beginning of numerous TCM healthcare facilities, clinics, and educational initiatives across Europe and beyond. Additionally, In 2009, the European Commission spearheaded the inception of the Good Practice in Traditional Chinese Medicine Research in the Post-genomic Era (GP-TCM) (Uzuner et al., 2010). This strategic initiative aims to establish guidelines for conducting research in Traditional Chinese Medicine (TCM), focusing on advocating for and advancing research standards within this domain (Uzuner et al., 2010; Uzuner et al., 2012). The GP-TCM consortium aims to provide best practices and coordinate safety studies to enhance the efficacy of Chinese medicine through the exchange of experiences and expertise across disciplines, facilitating collaboration between clinicians and scientists (Uzuner et al., 2012).

The core objective of the GP-TCM consortium revolves around harnessing functional genomics technology to establish deeper connections between TCM formulations and their clinically relevant biological functionalities. By doing so, the consortium endeavors to elucidate and substantiate the scientific merit of TCM comprehensively and functionally. This pursuit aligns with the broader mission of bridging the gap between traditional Chinese medical practices and contemporary scientific understanding, fostering a more holistic and evidence-based approach to healthcare in Western countries (Uzuner et al., 2012).

### 4.2 Cultural contrasts in oral prescription utilization between Eastern and Western medical traditions

Throughout the world, diverse cultures have cultivated unique frameworks of science, each giving rise to distinct medical practices aimed at promoting community health (Zhao et al., 2021). Healthcare systems worldwide face increasing costs and demands amid the challenges of a rapidly expanding global population (Jain et al., 2023). In addressing these issues, integrating Chinese and Western medicine presents a promising avenue for resolution (Van Der Greef et al., 2015). Hence, this section delves into the cultural and theoretical variances in prescription utilization between these two medical paradigms.

In Western medicine, treatments typically focus on a singular active ingredient, often derived from plants, and involve the selection of potent compounds to target specific protein targets. Prominent examples include acetylsalicylic acid, extracted from willow trees and employed for pain and fever management (Vane and Botting, 2003; Arif and Aggarwal, 2024). In contrast, prescriptions rely on combinations of herbs blended into mixtures to foster potent therapeutic synergies, thereby promoting disease management through intricate interactions among the herbs. For instance, the Gegen-Qinlian decoction (GQD), utilized for addressing diarrhea and fever, consists of Puerariae Lobatae Radix, Scutellariae Radix, Coptidis Rhizoma, and Glycyrrhizae Radix et Rhizoma Praeparata cum Melle (Lu et al., 2021).

The absence of acknowledgment of Chinese herbal medicine (CHM) within Western medical circles stems from cultural disparities in medical practice. For example, in pharmaceutical quality control, assessing herbal medicines requires consideration of genotype, whereas the evaluation of synthetic medicines focuses on chemical structure (Shaw et al., 2012; Samuni et al., 2013). Despite the global popularity of CHM and its endorsement by a significant portion of the population, the explanation of complex molecular mechanisms remains a barrier hindering its acceptance within the framework of Western medicine (Chen et al., 2016). One primary obstacle is the stringent regulatory requirements for CHM ingredients in Western countries, such as mandating proof of at least 30 years of safe traditional use (Dobos et al., 2005). Additionally, the TCM complex composition further impedes their acceptance in the West (Tang et al., 2018).

Employing systems biology in the study of Chinese Herbal Medicine (CHM) offers a promising solution to bridge this gap and facilitate its acceptance in the Western medical environment (Xu et al., 2013). As a top technique in the current century, systems biology shares many similarities with Chinese medicine research methodology and thinking (Cai et al., 2018). Systems biology methods have unlocked numerous bioinformatics platforms, including genomics, proteomics, and metabolomics, providing powerful tools for studying the nature of symptoms and the efficacy of herbs in Chinese medicine (Li and Yang, 2008; Wang et al., 2021).

The application of systems biology in CHM research can yield a comprehensive understanding of the complex interactions between biological systems, thereby elucidating the mechanism and reliability of CHM treatments. By adopting a systems biology approach, researchers can delineate the molecular pathways underlying the therapeutic effects of CHM and furnish empirical evidence to enhance CHM’s efficacy. This scientific evidence enhances the credibility of Chinese medicine within the Western medical community and promotes its broader acceptance and integration into mainstream medical practice.

In summary, cultural contrasts in oral prescription utilization between Eastern and Western medical traditions underscore the need for cross-cultural understanding, adoption techniques, and integration of complementary healthcare approaches to optimize patient care and promote global health quality.

### 4.3 Revealing herbal keywords through our iterative approach in ancient texts

Classical Chinese medicine texts offer empirical insights for treating illnesses, boosting acceptance of TCM formulas as complementary therapies (Wang et al., 2013; Luo et al., 2022). Western countries increasingly use Chinese medicine for diseases (Liu J. et al., 2021). Chinese medicine uses concepts such as ‘hot’ and ‘cold’ for diagnosis and treatment. It dates back to the Han Dynasty’s Materia Medica (神農本草經) in 200–300 AD (Wang et al., 2016). Cold-Hot theory guided herbal preparation for millennia, classifying syndromes and herbs by hot, warm, cool, and cold properties (Covington, 2001). Ancient healers balanced herbs to treat hot and cold syndromes, using hot/warming herbs for cold-related syndromes and cool/cold herbs for hot-related syndromes (Wang et al., 2013).

Our *Apriori* algorithm findings support combining hot herbs (e.g., Angelica Sinensis) and cold herbs (e.g., Liquorice) in line with Chinese medicine theory; this suggests that cold-hot theory impacts herb interactions in formulas, aiding researchers in finding herb substitutions based on properties and exploring alternatives. However, our current program does not extract keywords based on this theory. We sought herbal substitution based on Chinese medicine assumptions, requiring further keyword classification development. *Apriori* results also revealed musk’s presence, a dried secretion from musk deer used in Chinese medicine, which historically caused population decline due to hunting (Liu K. et al., 2021).

The Materia Medica highlights the pain-relieving properties of musk. In ancient China, musk treated strokes, coma, and more. Musk is now scarce and costly, used more in European perfumes than medicine (Williams, 1999; Liu K. et al., 2021). Musk deer are classified into seven species, with only M. moschiferus not endangered, but its population is declining (Johnsingh and Manjrekar, 2013; Li et al., 2016). Chinese medicine sources musk from Moschus species, known for antibacterial, anti-inflammatory, and anti-cancer effects (Xu and Cao, 2016; Industry-leading clinical decision support, 2020). Scarcity led to synthetic musk use in cancer treatment (Xu and Cao, 2016); it emphasizes the challenge of finding alternatives from ancient records using association rules. Our iterative approach aids researchers by tagging keywords in these texts, facilitating correlation searches for novel medications.

We present an iterative approach for identifying keywords from ancient practices. The iterative keyword method helps to identify herbs mentioned in classical literature for use in various generative models. The language model based on archaic text automates text generation for medical professionals, researchers, and Chinese medical students who seek formulation information. Despite manual inspection, several keywords within ancient Chinese texts were still either not identified or labeled inaccurately. Moreover, the limited vocabulary of the corpus limits the variety of keywords in the language generation model. Updating the corpus could enhance efficiency. We recognize that our approach may ignore certain tags linked to formulas, herbs, and terminologies that could impact the accuracy of the formulas’ structural information. Efforts to improve accuracy will require validation and development. Accurate interpretation of Chinese medicine data necessitates consideration of herb dosage, combination, and TCM theory. As knowledge expands, more formula-related and herb-related term data will be essential. Our work pioneers data mining ancient texts to explore innovative formulas.

### 4.4 Conclusion

We structured a database of 33,823 keywords for subsequent AI training and data mining, containing significant information about Chinese herbal formulas and herbs. Our proposed iterative approach for classical TCM texts yielded insights into herbal combinations from diverse classical TCM literature. By cross-referencing KEGG disease pathways and Pu-Ji Fang herb pairs, we quantified literature instances via PubMed. Utilizing Chi-square or Fisher’s exact testing, we identified candidate herbs linked to herbal genetics. Through text mining, association rules, and LSTM generative models, we identified potential high-frequency herb substitution candidates based on co-occurring keywords from automated iterative approach annotations. We constructed the keyword network depicting herbal blends in formulations from classical text analysis findings. The herbal candidates identified provide potential substitutions for formulations featuring rare species components. In the future, we aim to enhance our understanding of herb-disease associations by incorporating additional data from expanded corpora, implementing a systematic validation process for our findings, and integrating links to external knowledge bases, such as genomics or proteomics.

## Data Availability

The original contributions presented in the study are included in the article/Supplementary material, further inquiries can be directed to the corresponding author.
