# Correction: AI-assisted literature exploration of innovative Chinese medicine formulas

**DOI:** 10.3389/fphar.2026.1788574

**Published:** 2026-02-09

**Authors:** Meng-Chi Chung, Li-Jen Su, Chien-Lin Chen, Li-Ching Wu

**Affiliations:** 1 Department of Biomedical Science and Engineering, National Central University (NCU), Jhong-Li City, Taiwan; 2 Education and Research Center for Technology Assisted Substance Abuse Prevention and Management, National Central University (NCU), Taoyuan, Taiwan; 3 Core Facilities for High Throughput Experimental Analysis, Department of Biomedical Sciences and Engineering, National Central University (NCU), Taoyuan, Taiwan; 4 IIHMED Reproductive Center, Taipei, Taiwan; 5 Tian Medicine Phamaceutical Company Ltd., Taipei, Taiwan; 6 School of Post-Baccalaureate Chinese Medicine, Tzu Chi University, Hualien, Taiwan; 7 Department of Health Promotion and Health Education, National Taiwan Normal University, Taipei, Taiwan

**Keywords:** text annotation tool, TCM, text mining, extraction, TCM LSTM generative model

There was a mistake in the Graphical Abstract and Figure 5C as published. The name of a referenced tool was incorrectly written with the Chinese character “德” instead of the correct character “得.” The incorrect spelling “世醫德效方” is corrected to “世醫得效方.” The corrected Graphical abstract and Figure 5 appear below.

**Graphical Abstract:**
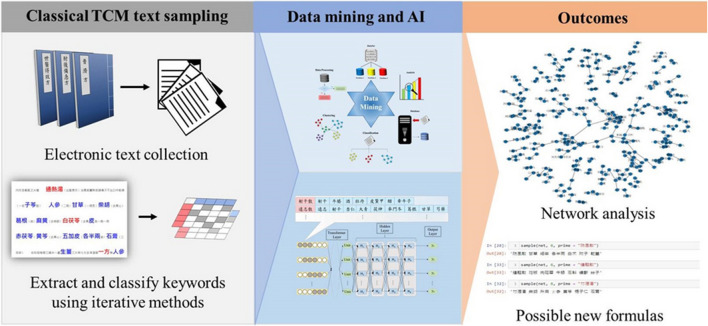


**FIGURE 5:**
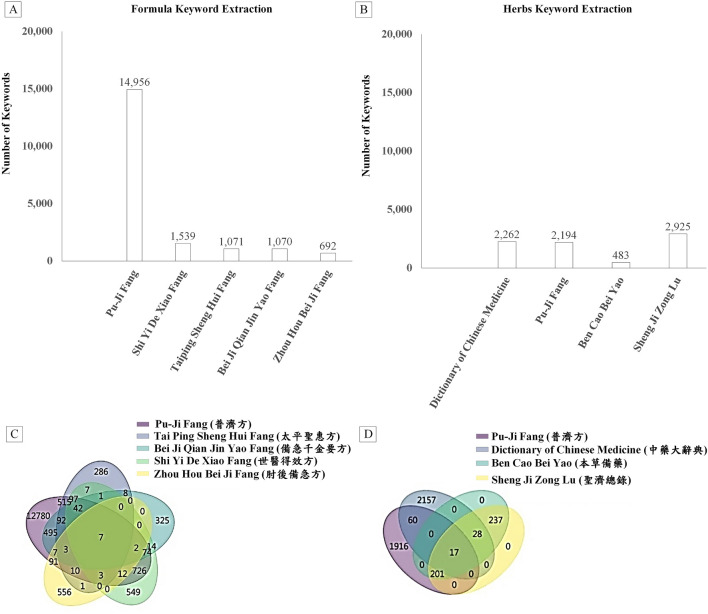
Histograms and union charts showing the number of keywords from various TCM texts. **(A)** Histogram depicting keyword counts in formulas; **(B)** Histogram illustrating keyword counts in herbs; **(C)** Union charts representing formula occurrences across various TCM texts; **(D)** Union charts displaying herb occurrences across various TCM texts.

In the published article, there was a spelling error in the name of a referenced tool. The character “德” was erroneously used instead of “得.” The incorrect spelling “世醫德效方” is corrected to “世醫得效方.”

A correction has been made to the Section 2 Materials and methods, 2.1 Data collection and corpus building, Paragraph Number 3.

“Ancient Chinese medicine e-books were sourced from Wikipedia, including Pu-Ji Fang (普濟方), Ben Cao Bei Yao (本草備要), Sheng Ji Zong Lu (聖濟總錄), Shi Yi De Xiao Fang (世醫得效方), Taiping Sheng Hui Fang (太平聖惠方), Bei Ji Qian Jin Yao Fang (備急千金要方), and Zhou Hou Bei Ji Fang (肘後備急方), for text analysis and keyword refinement.”

The original article has been updated.

